# COVID-19 and systemic sclerosis: Rising to the challenge of a pandemic

**DOI:** 10.1177/2397198320963393

**Published:** 2020-10-18

**Authors:** Christopher P Denton, Corrado Campochiaro, Cosimo Bruni, Oliver Distler, Annamaria Iagnocco, Marco Matucci Cerinic

**Affiliations:** 1 Division of Medicine, Department of Inflammation, Centre for Rheumatology and Connective Tissue Diseases, Royal Free Campus, University College London, London, UK; 2 Unit of Immunology, Rheumatology, Allergy and Rare Diseases (UnIRAR), IRCCS San Raffaele Hospital, Vita-Salute San Raffaele University, Milan, Italy; 3 Department of Experimental and Clinical Medicine, Division of Rheumatology, University of Florence, Florence, Italy; 4 Centre for Experimental Rheumatology, University Hospital of Zurich, Zurich, Switzerland; 5 Academic Rheumatology Centre, Università degli Studi di Torino, Turin, Italy

**Keywords:** COVID-19, lung fibrosis, pathogenesis, EULAR, scleroderma

## Abstract

COVID-19, caused by infection of the novel coronavirus SARS-CoV-2, has caused a pandemic of enormous impact that has challenged healthcare and political system throughout the world. This new health emergency has occurred on top of the usual burden of diseases, including systemic sclerosis, and has led to many unanticipated consequences. An early consequence of the pandemic was postponement of the Sixth Systemic Sclerosis World Congress that was recently completed as a successful virtual congress with more than 1000 delegates. In this article, we summarise the relevance and impact of COVID-19 from the perspective of systemic sclerosis. Shared concepts of pathogenesis are considered, and the relevant literature emerging about COVID-19 and systemic sclerosis summarised. The specific impact of this pandemic on delivery of optimal scleroderma care is considered, together with the broader effect on rheumatic and musculoskeletal diseases and the activities of European League Against Rheumatism. As the World continues to struggle against this new infectious disease, it is notable that expertise and growing understanding of systemic sclerosis has been able to help tackle COVID-19. Moreover, the essential adjustments to deliver clinical care and establishment of new ways of working due to the pandemic have offered potential avenues for future improvement in systemic sclerosis care.

## Introduction

The global impact of COVID-19 has been the defining societal and medical struggle of the 21st century and has justifiably received top priority for healthcare systems around the globe. This has inevitably affected the resources and facilities available for management of other long-term diseases. This article is based upon a series of presentations given at the Sixth Systemic Sclerosis World Congress, that took place as a ‘virtual’ event on 12–14 July 2020. This was a live broadcast session of the congress that focused attention on important aspects of COVID-19 that are relevant to scleroderma.^1^ First, form a mechanistic viewpoint, there are shared mechanisms and insights relating to the pathogenesis of both COVID-19 and systemic sclerosis (SSc, scleroderma). Next, there is an emerging literature on the specific topic of COVID-19 in SSc that has clear and practical relevance to all scleroderma patients. In this context, it is not surprising that clinical overlap exists and that there are potential lessons from scleroderma management applicable to COVID-19. There has been a devastating impact on delivering care for scleroderma as the healthcare resources were necessarily redirected towards COVID-19. To improve the evidence base for research and clinical care, there are ongoing efforts to collect systemic data that will fuel and inform future research and management, and this includes initiatives of World Scleroderma Foundation (WSF) and European Scleroderma Trials and Research (EUSTAR). Finally, systemic sclerosis can be seen in a broader context and the efforts of European League Against Rheumatism (EULAR) across the rheumatic and musculoskeletal diseases will underpin the future mitigation of the detrimental impact of COVID-19 in this space and harness expertise and resources to help move forward and embrace the opportunities that this unprecedented challenge has presented.

## Pathophysiology of COVID-19 and similarities with SSc

**Figure 1.**
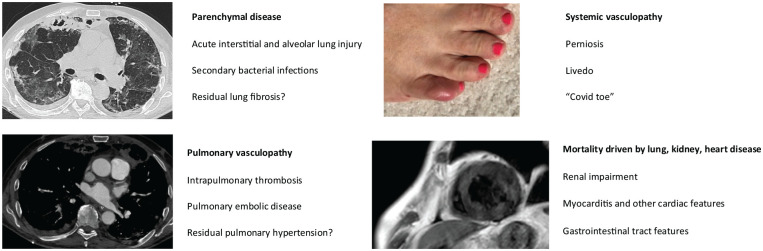
Shared clinical features of systemic sclerosis and COVD-19. There are shared clinical features between systemic sclerosis and COVID-19, including mixed patterns of parenchymal and vascular lung disease, myocardial abnormalities with inflammation and systemic peripheral vasculopathy as evidenced by lesions of the toes, in this case with perniosis (so-called ‘Covid toe’). All clinical images are from patients with COVID-19.

Cardinal pathogenic processes are shared between COVID-19 and SSc albeit with distinct triggering mechanisms. It is likely that in SSc there are multiple triggering events over several years that ultimately trigger the disease, and explain how an individual may move from Raynaud’s into early SSc, and then established disease. In COVID-19, the key factor is virus exposure and subsequent infection. However, where there may also be great similarities is the outcome and pattern of disease that develops once it is present.^2^ There have now been clear studies defining the genetic susceptibility to SSc and to different patterns of disease and outcomes. In the same way, similar mechanisms may determine severity and outcome from COVID-19. Support for this comes from the observation that susceptibility genes for SSc are especially within the innate and adaptive immune system, particularly when considering common variant single nucleotide polymorphisms (SNPs) and polymorphism, and so it is likely that these genes are also relevant to infective disease.^3^ In addition, there may be specific genetic susceptibility linked to genetic variation in the viral receptor protein ACE2.^4^ The fact that they are shared across other immune mediated diseases adds weight to that argument. With that in mind, it is notable that key demographic groups that have higher mortality or worse outcome from SSc are also over-represented in COVID-19 deaths. The aetiopathogenetic framework for COVID-19 and SSc, highlighting the likely role of host factors that determine clinical phenotype and outcome, is compared in Figure 1.

**Figure 2.**
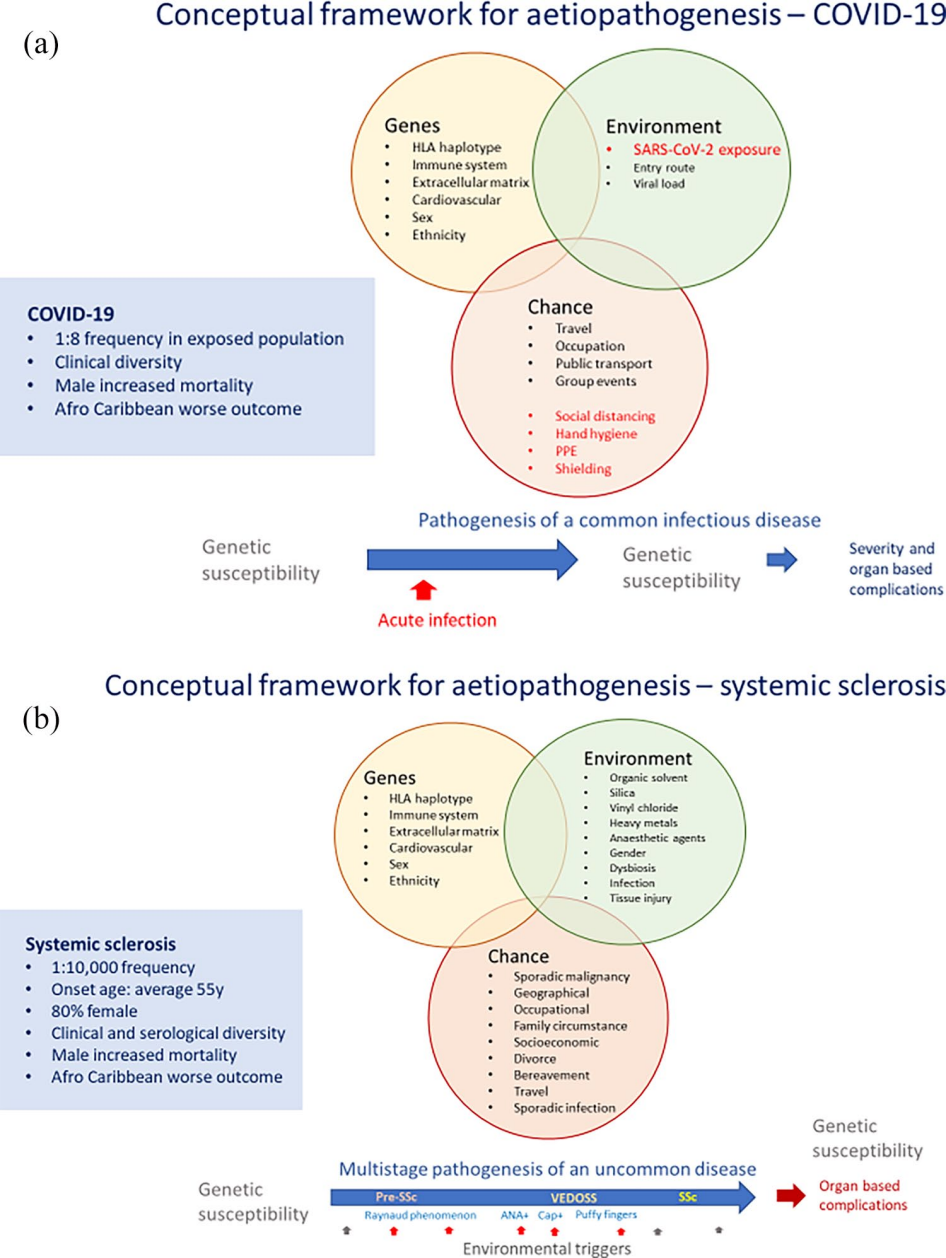
Conceptual framework for aetiopathogenesis of systemic sclerosis and COVID-19: The development of clinical disease depends upon three factors. Susceptibility of the individual, the appropriate environmental trigger or series of triggers that lead to disease and random chance that determines whether the trigger meets the host at an appropriate time to cause disease. (a) For COVID-19 clearly the trigger is SARS-CoV-2 the trigger, but a multitude of host factors determine the timing, dose, and pattern of exposure. Host factors are highly likely to be the major determinant of disease phenotype and clinical outcome. (b) Analogy can be drawn with a complex chronic disease like systemic sclerosis. Here multiple triggers over time are likely to be required and lead to progression from pre-disease state to established diagnosis. Host factors are also likely to be major determinants of disease phenotype, severity, and outcome. It is possible that immunogenomic factors determining susceptibility to tissue damage and poor outcome are shared between the autoimmune landscape of SSc and the virally triggered immuno-inflammation of COVID-19.

While it might be considered that a rare fibrotic connective tissue disease such as SSc might have little in common with a very frequent infectious disease caused by the novel coronavirus SARS-CoV2, there are likely to be valuable lessons and insights from comparing the aetiopathogenesis of these disparate conditions. Shared features are summarised in Figure 2 and discussed below.

Thus, both conditions clearly have environmental triggers, but in COVID-19, this is exposure and infection by SARS-CoV2 and in SSc generally the triggers are less clear. Some obvious triggers of SSc and scleroderma-like diseases have emerged. Thus, it seems that the triggers in SSc likely occur over many years in a multistep process, whereas in COVID-19 this is a universal and proximal event to disease.

However, once the disease begins it seems likely that host factors are the primary determinant of clinical manifestation, severity, and outcome. In this context, there are far more similarities between the two diseases. The pattern of organ involvements and prominence of vasculopathy is clear as is the major determinant of outcome from cardiorespiratory complications. It is likely that answers about the specific genetic, immunogenomic, and comorbid factors important for COVID-19 will be elucidated far more rapidly than has been possible for SSc due to the global prevalence of the disease, our current knowledge base in these areas and the enormous medical and scientific capital that is being invested in discovery science, epidemiology, and clinical research into this global pandemic.

## Shared pathobiology of COVID-19 and systemic sclerosis

Even though the fundamental aetiological triggers are different, there are many similarities in likely pathological mechanisms between SSc and COVID-19, and this is highlighted by the growing literature describing fundamental science studies in COVID-19. This has the potential to advance understanding of pathogenesis of both diseases and draw parallels in other infectious diseases and chronic multisystem disorders.

First, it is useful to consider the fundamental pathogenetic mechanisms in SSc that include vasculopathy, immune-inflammation with release of cytokines, and organ-based fibrosis. There have been pertinent studies in each of these areas recently published in relation to COVID-19 and some are considered below.

### Vasculopathy

Although first recognised as a respiratory illness leading to viral pneumonia and acute respiratory distress syndrome (ARDS), it rapidly became apparent once the pandemic reached European centres that a major component of the disease related to vasculopathy. This was apparent in terms of the prediction for thromboembolic disease, including pulmonary emboli and evidence of intravascular thrombosis at many sites. It may be a key factor in the hypoxia that is characteristic of COVID-19. Endothelial activation and damage are prominent, and these have been shown clearly in recent research studies, including the excellent work of Ackermann and others. Interestingly, there was also considerable clinical evidence of systemic vasculopathy, including acrocyanosis and severe digital insufficient and perniotic lesions of the extremities (including the well described ‘Covid toe’).^5^ Interestingly, vascular therapies and anticoagulation appeared to be important in management and may contribute to improved outcomes for more recently managed cases. Other relevant mechanism for vasculopathy include a direct role from viral infection via the ACE2 protein expressed on endothelial cells and coagulopathy, and complement mediated damage.^6^ The consequence of these changes has been recently demonstrated at the level of the microcirculation using nailfold capillaroscopy and showing reduced capillary density and associated morphological changes, and haemorrhage that are a hallmark of early or active SSc.^7^ The importance of endothelial dysfunction has been highlighted in elegant studies comparing COVID-19 lung histology with that from influenza virus–associated ARDS which highlighted the prominence of endotheliitis in COVID-19.^8^

### Cytokine release syndrome

Perturbation of the normal immune response and cytokine production is central to the development of chronic immune-mediated diseases such as systemic sclerosis. This is likely to reflect excessive immunological activation and cytokine driven inflammation together with a failure of the switch off signals that normally accompany appropriate resolution of inflammation and facilitate normal repair and regeneration. This may be driven by activation of pathways via the innate immune system, possibly resulting from damage-associated molecular patterns (DAMPs) from dying cells leading to excessive inflammatory responses or activation of interferon driven pathways, or from stimulation of the adaptive immune system by viral antigens.^9^ In the context of COVID-19 both processes are likely to occur, especially in the subgroup of cases that develop severe lung inflammation and have a poor outcome. This may result from excessive release of major pro-inflammatory cytokines as have been observed in other clinical contexts such as after cellular therapy for cancer (chimeric antigen receptor (CAR)-T treatment) and in conditions such as hemophagocytic lymphohistiocytosis (HLH) (macrophage activation syndrome (MAS)). In the context of COVID-19, it may be that intrinsic factors relevant to the immune system and inflammation (such as immunogenomic background) determine how effective the immune response to the primary viral infection is at elimination the virus and limiting secondary tissue damage. It seems highly likely that similar factors may determine clinical phenotype, outcome and patterns of disease and organ involvement in systemic sclerosis. The degree of ongoing antigen driven or antigen independent immune-inflammation may be central. However, although these mechanisms may overlap, there are also likely to be different. This is likely to be apparent when more targeted approaches to cytokine or cellular targeting are applied and the results of ongoing clinical trials of targeted therapies in COVID-19 are likely to be informative. It is notable that such therapies may have major benefit in SSc but appear to only target subsets or stages of the disease (e.g. FocuSSced and ASSET randomised clinical trials), and this may be the case for COVID-19 or explain why broader approaches such as steroids may be needed or be superior to very precise targeted treatments (e.g. tocilizumab (TCZ)).

Similar shared mechanisms may be important in mediating endothelial activation and injury, and studies of complement and coagulopathy are especially interesting as these may offer a mechanistic link for the inflammation of COVID-19, and the profound vasculopathy and especially the thrombotic manifestations that seem to be very important determinants of clinical outcome in the lung and other organs.

### Fibrosis and scarring

Systemic sclerosis is a prototypic fibrotic disease and so it is entirely plausible that the shared pathogenic processes linked to vasculopathy and immuno-inflammation may lead to scarring of damaged organs. This may be especially relevant in the lungs where there can be less capacity for tissue repair and regeneration that occurs in some other organs. This is relevant because in the future, fibrosis may become a recognised manifestation of COVID-19. In other novel coronavirus infections such as SARS1 and Middle East respiratory syndrome (MERS), up to 25% of cases have developed interstitial lung fibrosis and it is already apparent that residual computed tomography (CT) abnormalities may persist after the initial acute infection in COVID-19.^10^ The mediators of fibrosis after acute lung injury in COVID-19 will almost certainly include key mediators in SSc and could include transforming growth factor (TGF-β), interleukin-6 (IL6), connective tissue growth factor (CTGF) and downstream pathways that lead to increased activation of myofibroblasts, excessive extracellular matrix (ECM) deposition or failure of appropriate ECM breakdown as the lung injury resolves. It is notable that parallels are likely between pathogenesis of COVID-19 and the early progressive phase of other forms of interstitial lung fibrosis.^10^ The longer-term implications will need to be determined in future studies, and there may be shared features between those individuals that later develop lung fibrosis after COVID-19 and the cases of SSc that develop significant or progressive interstitial lung fibrosis. This could also have potential implications for therapy.

## SSc-COVID: the voice of the literature

The concerted efforts of medical teams dealing with COVID-19 has led to a growing medical and research literature describing the disease, and providing insight into the clinical challenges and management approaches that are being used and evaluated. This includes a significant literature that specifically relates to SSc. One of the first relevant papers is the recommendation document from the World Scleroderma Foundation that addressed the occurrence and management of COVID-19 in systemic sclerosis.^11^ This provided a timely summary of the collective guidance from many experts and professional bodies that related to rheumatic disease and the risk and management of COVID-19. Following on from this publication, there have been several case reports and series that are now published, and some of the key articles, including laboratory, clinical and therapy features, are summarised in Table 1. In addition, there has been a systematic review of the available literature related to COVID-19 in systemic sclerosis and the publication of this important paper is eagerly awaited.^18^ Current available evidence lacks standardised data collection approach and, therefore, almost no conclusion can be derived from the literature so far, in particular regarding COVID-19-related organ involvement, change in background SSc manifestation and response to treatment. Therefore, important open questions will be addressed by the analysis of a larger systematically collected dataset from COVID-19 cases, including the emerging reports from the various COVID19-SSc registries. These questions include identification of factors that may protect or mitigate COVID-19 infections or modulate the outcome. This may include treatments that are already in use as therapies for SSc. In addition, risk factors that predict good or poor outcome in the context of SSc may be identified. It is intriguing to speculate whether these may be the same factors that predictor risk of organ-based disease and death in SSc.^19^ Another open question is whether the pattern of disease and frequency of manifestations are different for SSc-COVID than in the general population. This might differ based upon the potential clinical and pathogenesis overlap outlined in this article. Finally, the impact of SSc and current candidate therapies on COVID-19-related outcomes, including intensive care unit admission and mortality, should be addressed in future observational cohort studies and can be extrapolated from ongoing clinical trials that evaluate drugs that are also being tested or are in routine clinical use in SSc, such as corticosteroids and TCZ.

**Table 1.** Summary of key literature for COVID-19 in systemic sclerosis.

Author	Patient age	Patient gender	Ongoing immunosuppressive SSc for treatment	Subset	Presence of pre-existing ILD	Auto-antibody positivity	Ventilation support needed	Anti-cytokine therapy used against COVID-19	Death
Avouac et al.^12^	71	M	RTX + MTX + CCS Yes	dcSSc	No	RNA pol III	Yes, non-invasive	No	No
	84	F	Yes RTX + CCS	lcSSc	No	Unk	No	Yes, Anakinra and TCZ	Unk
	44	F	RTX + MTX + CCS Yes	lcSSc	No	RNA pol III	Yes, non-invasive	No	Unk
Cheng et al.^13^	79	M	Yes CCS	Unk	Unk	Unk	No	Yes, TCZ	No
Favalli et al.^14^	32	F	Yes HCQ + RTX	Unk	Yes	Unk	Yes, invasive	Yes, TCZ	Yes
Mihai et al.^15^	57	F	Yes TCZ	Unk	Yes	ATA	No	Yes, TCZ	No
Moiseev et al.^16^	65	F	Unk	dcSSc	Yes	Unk	Yes, both invasive and non-invasive	No	Yes
	66	F	Unk	Unk	No	Unk	Unk	Unk	Yes
Zen et al.^17^	54	F	MMF	Unk	Unk	Unk	Yes, Low flow oxygen	No	No

ATA: anti-topoisomerase; dcSSc: diffuse cutaneous systemic sclerosis; lcSSc: limited cutaneous systemic sclerosis; MMF: mycophenolate mofetil; MTX: methotrexate; ARA: anti-RNA polymerase III antibody; RTX: rituximab; TCZ: tocilizumab; unk: unknown.

## Impact of COVID-19 in delivering a scleroderma service

There have been many ways that COVID-19 has affected SSc and its management. In one of the author’s institutions at the Royal Free Hospital in London as the number of COVID cases started to increase from mid-March2020 and all routine clinical activity was cancelled. The hospital essentially moved to being almost exclusively dedicated to COVID care and most of the medical and nursing staff were redeployed to provide care. It also led to cancellation of all routine outpatient visits and inpatient admissions and cancellation of the routine monitoring investigations such as cardiorespiratory testing that are a cornerstone of modern SSc management. This transformation was documented in an excellent British Broadcasting Corporation (BBC) documentary that has been broadcast since the pandemic.^20^

As discussed elsewhere in this article, there have been important parallels between SSc and COVID-19 that meant that those familiar with managing SSc were well placed to move into COVID care and this has happened around the world. Rheumatologists have been treating immunoinflammatory disease with vasculopathy and complications affecting the lungs, heart and kidneys, and this made the disease familiar. Moreover, frequent involvement of the gastrointestinal tract and emerging appreciation of the generalised nature of vasculopathy and the skin manifestations of COVID^21^ added to this perspective.

In addition, familiarity with defining ceilings of care and the challenge of multisystem disease requiring intensive care support in the context of co-morbidity was a familiar challenge for those who normally care for SSc. This made them valuable members of the multidisciplinary team and as familiarity and expertise grew, it was possible to translate some treatment approaches from SSc into COVID including use of anti-cytokine treatment. This was facilitated by the familiarity of rheumatologists with immunomodulatory and anti-cytokine treatments and with an appreciation of trial design. Interestingly, emerging treatments for SSc were often proposed as candidate therapies for COVID-19. This includes IL6 receptor blockade, CB2 endocannabinoid agonists and angiokinase inhibition by nintedanib. The beneficial impact of prostacyclin agonists such as iloprost has been suggested, and this is supported by a case series that suggested benefit for reduced oxygen requirement in cases as well as possible improvement in the digital ischaemia that has been recognised as a component of some cases of COVID-19.^22^

One of the consequences of having rheumatologists and other SSc experts join the medical service managing COVID-19 was the harnessing of expertise across these two diseases. Through the pandemic, it became apparent that there are many similarities between the diseases that reflected shared pathogenic mechanisms and disease biology.

## The EUSTAR registry for COVID-SSc cases

It was appreciated very early in the pandemic that there was a pressing need for systematic data collection to understand better the impact of COVID-19 in the context of a rare disease like SSc. This led in March 2020 to the EUSTAR/WSF initiative to capitalise on the established and mature EUSTAR registry of more than 17,000 cases from many centres and use this for COVID data collection. The goal is to harmonise and link with other COVID-19 initiatives but allow collection of more SSc-specific details. This will be especially relevant to the management of COVID-19, and to provide insight into both diseases based upon the factors outlined above concerning shared pathogenesis and overlapping clinical manifestations. In addition, it has important implications for future management and approaches to shielding and protecting high-risk patients and issues such as continuation or interruption of immunosuppression, and other potential therapies for SSc.

Analysis of the registry data will address key topics highlighted in the literature review section above: key aims of the EUSTAR registry are to document the course of COVID-19 in patients with SSc and to identify any SSc-specific risk factors for poor outcome. It will also show which treatments used in COVID-19 cases with SSc and may even suggest possible treatment effects, although conformation in future controlled trials is likely to be needed.

The SSc registry represents an important collaborative effort with engagement of other international initiative including the EULAR COVID-19 and the global alliance COVID-19 registry.^23^ Both of these registries have agreed to include the SSc-specific questionnaire to obtain additional relevant data that can be incorporated into the EUSTAR/WSF registry or provide external validation. In addition, the broader rheumatic disease registries will provide invaluable data to benchmark and compare SSc cases, and define whether there are disease-specific aspects for SSc that are not shared by other rheumatic and musculoskeletal diseases.

To date, there have been over 100 patients registered, but this number is likely to substantially increase once data from collaborating projects is included. There is of course care to avoid double recording of cases that may have been included in more than one register.

The outcomes and full analysis of the data from this valuable registry will be included in future substantive publications.

## EULAR activities in the COVID era

There has been a major impact on almost all aspects of life including global rheumatology and patients and professionals as well as governments. The timing of the first wave, as it spread through major European countries, made it very apparent that travel restrictions and deployment of staff to the COVID frontline would have a major effect on medical conferences. One of the first affected for rheumatology was the Sixth Systemic Sclerosis World Congress that had originally been scheduled for 5–7 March 2020. A few days ahead of the congress, despite late approval from local health authorities and government, it was decided that the meeting had to be postponed. This was an enormously challenging decision for the Congress leadership but one that now looks prescient. The World Congress was redeveloped as a virtual event and has just concluded. It was a great success. In part, this was possible because it was able to draw upon the experience and expertise of those involved in the 2020 EULAR Congress. That was due to take place in June 2020, and so there was an early appreciation hat the usual template could not be followed. Therefore, EULAR Congress became completely virtual. An online recorded and live congress programme was developed and there were over 18,000 delegates. This represents one of the first and largest such medical congress in the COVID era. Nevertheless, much smaller than the face-to-face conference. Lessons learned will be incorporated into future events, and there is great hope for future face-to-face meetings that may also incorporate the best of the virtual space to increase accessibility and reach of the congress.

Other key EULAR activities include the development of EULAR recommendations for required minimum distributions (RMDs) in COVID-19 and discrepancy with American College of Rheumatology (ACR) recommendations. A COVID Task Force conducted this work and these updated RMD recommendations have developed 5 principles and 13 recommendations.^24^ As discussed above, a EULAR COVID-19 Database has been formed collaboration with PRESS and with contributions from different EULAR countries. There are currently more than 2400 patients in the registry.

Practical guidance for patients with rheumatic and musculoskeletal diseases reflecting the importance of patients including and communication has been developed. This includes educational resources including webinars and involvement of PARE on behalf of patients. Information to help providers ensure that as services are re-opened, they are COVID safe and COVID secure. There is also a longer-term effort focused on mitigation of the impact of predicted economic recession for RMD. It is appreciated that while all current live events and offerings are cancelled, postponed, or becoming virtual this is proving successful and will feed into future planning to address the goals of EULAR even when the pandemic has passed.

Finally, research harnessing expertise form rheumatology scientific and clinical community to improve assessment, treatment and understanding of COVID-19 using the EULAR COVID-19 Task Force.

## Conclusion and future perspective

At the time of writing, we are still facing an enormous challenge from COVID-19. In most European countries, the first wave of the pandemic appears to have passed but as public health measures are relaxed and communities and countries move towards a ‘new normal’, case numbers are rising. This appears to be driven by more localised outbreaks than in the first period of exponential spread within the community. It is a profound hope that the systems are now in place to allow these outbreaks to be identified and controlled, without the need to revisit severe national lockdowns. However, the future remains uncertain. What is clear is that serious diseases like systemic sclerosis much now receive their deserved attention so that their management can be restored. It is reassuring that initial results do not suggest a major negative impact of COVID-19 on the SSc population, although this may have been masked by effective shielding of SSc patients in the first wave. Until the number of new cases falls substantially, effective proven treatments become available and/or a vaccination is developed that prevents or attenuates COVID-19, it will remain a major priority for healthcare around the globe.
